# A Channel-Independent Anchor Graph-Regularized Broad Learning System for Industrial Soft Sensors

**DOI:** 10.3390/e28030274

**Published:** 2026-02-28

**Authors:** Zhiyi Zhang, Mingyi Yang, Cheng Xie, Zhigang Xu, Pengfei Yin

**Affiliations:** 1 Shenyang Institute of Automation, Chinese Academy of Sciences, Shenyang 110016, China; zhangzhiyi@sia.cn (Z.Z.); xiecheng@sia.cn (C.X.); zgxu@sia.cn (Z.X.); 2 University of Chinese Academy of Sciences, Beijing 100049, China; 3 Xi’an North Huian Chemical Industries Co., Ltd., Xi’an 710302, China

**Keywords:** soft sensor, broad learning system, quality prediction, channel independence, manifold regularization, anchor graph

## Abstract

To address the nonlinear dynamics and strong multivariate coupling inherent in complex industrial data, while overcoming the high computational costs and deployment challenges of deep learning, this paper proposes a Channel-Independent Anchor Graph-Regularized Broad Learning System (CI-GBLS). First, a Channel Independence (CI) strategy is introduced: by constructing physically isolated feature channels, multivariate inputs are orthogonally decomposed, enabling the model to mine the intrinsic temporal evolutionary patterns of each variable. Building upon this, enhancement nodes are constructed using Radial Basis Functions (RBFs) to capture nonlinear dynamics; moreover, RBF cluster centers are reused as graph anchors to design an efficient manifold regularization algorithm. This algorithm embeds the intrinsic geometric structure of the data into the learning objective via reduced rank approximation, thereby guiding output weights to explicitly reconstruct spatial coupling relationships while preserving manifold consistency. Experimental results on the IndPenSim process demonstrate that CI-GBLS effectively balances prediction accuracy and efficiency. It completes training within seconds, validating its effectiveness for complex time-series data and offering an efficient solution for real-time, high-precision industrial modeling.

## 1. Introduction

In industrial production, the real-time measurement of quality parameters is essential for ensuring safe, stable operations and facilitating process optimization. However, certain quality parameters in practice cannot be measured directly by hardware sensors, necessitating reliance on offline methods such as laboratory assays. The resulting measurement latency often leads to delayed responses in the production process [1]. Soft sensor technology enables the online prediction of quality parameters with high speed and accuracy, making it an effective approach for monitoring quality variations. Consequently, it has been widely applied in chemical, metallurgical, and other industrial sectors [2,3,4,5,6].

Soft sensor modeling methods can generally be classified into mechanism-based modeling and data-driven modeling. Mechanism-based modeling requires an in-depth understanding of the chemical or physical processes within the industrial system and involves constructing complex mathematical models. In practical applications, developing precise mathematical models often entails extremely high complexity [7]. In contrast, data-driven modeling establishes predictive models using large volumes of process data without requiring detailed knowledge of internal mechanisms, offering faster model development and higher prediction accuracy. Early machine learning methods, such as Support Vector Machines (SVMs) and Artificial Neural Networks (ANNs), have been applied in industrial processes [8,9,10,11]. Nevertheless, complex industrial data often exhibit strong nonlinearity and coupling characteristics, which limit the effectiveness of shallow machine learning methods in feature extraction. Consequently, deep learning has been introduced into the soft sensor field due to its superior feature extraction and nonlinear modeling capabilities; for example, Long Short-Term Memory (LSTM) networks and various attention-based approaches have demonstrated outstanding performance [12,13,14,15,16]. However, deep learning architectures typically involve long training times, high computational costs, and reliance on massive, labeled datasets. Moreover, real-world production processes often exhibit nonstationary characteristics, such as environmental fluctuations and operational condition drift, which can degrade model accuracy. In such scenarios, the high retraining overhead of deep neural networks makes it difficult to rapidly adapt to the changing production environment.

The Broad Learning System (BLS), proposed by Chen and Liu [17], offers an alternative paradigm to overcome the limitations of deep learning. Unlike deep learning, which relies on deep architectures to approximate complex functions, BLS achieves mapping capability by expanding the network width. Structurally, input data are mapped into feature nodes, which are then nonlinearly expanded into enhancement nodes, with all nodes directly connected to the output layer. BLS eliminates the need for gradient-based iterative optimization; instead, output weights can be obtained algebraically, significantly reducing both training time and computational costs. To further enhance the feature extraction capacity of BLS, various structural improvements and extensions to the standard BLS have been proposed [18,19,20,21].

Ye et al. [22] introduced a deep cascaded broad learning system that employs cascaded feature and enhancement node layers, enabling more thorough extraction of effective information; however, the deeper structure decelerates training speed. Liu et al. [23] proposed a stacked broad learning system that vertically stacks multiple BLS modules and allows new modules to learn residuals, achieving superior fitting capability. Nevertheless, the underlying feature extraction mechanisms in these methods still rely on BLS’s random mapping strategy, limiting the model’s ability to capture nonlinear characteristics of variables.

In response, some researchers have focused on improving the feature mapping mechanism of BLS. For example, Zhao et al. [24] proposed an adaptive stacked approximate kernel broad learning system that projects feature nodes into a high-dimensional kernel space using approximate kernel techniques, demonstrating superior performance in handling batch process data characterized by strong nonlinearity and time-varying dynamics. Wan et al. [25] introduced a Dynamic Attention-based Broad Random Forest (DA-BRF) model, which substitutes Random Forests for feature nodes in BLS and incorporates an attention mechanism to dynamically capture time-varying correlations among variables, effectively improving prediction accuracy in complex industrial processes. In a recent study, the BLS architecture was improved to capture the nonlinear dynamic interactions among variables [26]. Although these improved methods have achieved significant progress in feature mapping mechanisms, most focus on exploring features by modifying mapping functions or adding attention modules, often overlooking the latent local manifold structure among data samples. To address this issue, Zhao et al. [27] recently proposed a broad learning system incorporating a stacked Laplacian autoencoder. This study constructs a deep autoencoder with manifold constraints at the front end of BLS for feature extraction, demonstrating that preserving local geometric consistency of data during feature learning is crucial for enhancing industrial quality prediction performance. However, this method essentially embeds manifold learning as a separate pre-training module into the feature extraction stage, which, while effective, increases the complexity of the model architecture. Furthermore, research in the field of process monitoring demonstrates that manifold constraints effectively preserve the local geometric consistency of industrial data, thereby significantly enhancing model performance in complex environments [28].

Moreover, when handling high-dimensional multivariate industrial data, the aforementioned broad learning variants have inherited the fully connected input processing strategy (Channel Dependence), which concatenates all process variables and feeds them into the network. However, variables in real industrial processes often exhibit significant multi-rate characteristics and heterogeneous dynamic differences. The fully connected structure forces all variables to share the same parameter space, making it difficult to accommodate temporal features with different response frequencies. This inevitably leads to mutual interference among features and spurious couplings, thereby limiting the model’s generalization capability under complex operating conditions.

Recent studies in time series forecasting have found that a Channel-Independent (CI) strategy, which decouples multivariate inputs and processes them independently, often achieves superior predictive performance. Specifically, the DLinear model proposed by Zeng et al. [29] decomposes variables into independent channels for simple mapping in multivariate analysis, effectively eliminating invalid interactive features and achieving higher prediction accuracy with a more compact model structure. The PatchTST model introduced by Nie et al. [30] applies the CI strategy within a Transformer architecture, allowing all independent channels to share model weights, thereby effectively avoiding noise interference from irrelevant channels and significantly enhancing generalization ability in long-sequence forecasting. Han et al. [31] further theoretically demonstrated that constraining inter-channel interaction capacity can effectively prevent the overfitting trap in high-dimensional spaces, thus achieving better accuracy on test sets.

Although these studies primarily target long-term time series forecasting tasks, their decoupling principle offers valuable insights. Employing the channel independence strategy enables models to first focus on mining intrinsic temporal patterns from each individual variable, ensuring accurate temporal information capture. Subsequently, the network’s output layers can perform inter-channel information coupling and recombination. This modeling paradigm, characterized by first extracting high-purity temporal features and then fusing spatial correlations, helps reduce complexity during the feature extraction phase and provides a more precise feature foundation for final multivariate fusion prediction.

To guide the model output to adhere to the intrinsic manifold distribution of data for enhanced prediction consistency and aiming to achieve decoupled modeling and accurate reconstruction of temporal dynamics and spatial coupling in high-dimensional industrial data, this paper proposes a Channel-Independent Anchor Graph-Regularized Broad Learning System (CI-GBLS). Structurally, CI-GBLS introduces a Channel-Independent (CI) strategy into soft sensor modeling. By utilizing the CI strategy to construct independent feature mapping and enhancement channels for each process variable, the complex multivariate modeling task is decomposed into multiple univariate sub-tasks, compelling the model to focus on mining the intrinsic temporal features of each variable itself. Simultaneously, the model adopts Radial Basis Function (RBF) kernel mapping instead of traditional randomly generated enhancement nodes, utilizing K-Means clustering to adaptively determine kernel centers for more robust fitting of nonlinear features. To exploit the data manifold structure without sacrificing the efficient solving advantage of BLS, this paper incorporates an Anchor Graph-based manifold regularization term into the objective function, employing the kernel centers obtained from K-Means clustering as anchors. By leveraging Reduced Rank Approximation techniques, the graph Laplacian calculation is transformed into efficient matrix algebraic operations. This ensures linear computational complexity while embedding the global geometric constraints of data into the model as prior knowledge, guiding the output weight matrix to strictly adhere to the physical consistency of industrial processes during the reconstruction of coupling relationships. Ultimately, through the synergy between the linear weighting mechanism of the output layer and the aforementioned manifold regularization term, the model achieves adaptive reorganization of inter-variable information and obtains optimal output weights with global consistency constraints within an analytical solution framework.

The main contributions of this paper are summarized as follows:

1. A Channel-Independent Broad Learning architecture is proposed. In response to parameter redundancy and spurious correlations caused by fully connected modeling of industrial multivariate data, this architecture maximizes the extraction accuracy of univariate temporal features via a decoupling-reconstruction strategy and explicitly recovers spatial correlations among variables through global weights in the output layer. This architecture reduces the model’s computational complexity from $O\left(N^{2}\right)$ to $O\left(N\right)$, significantly enhancing feature extraction efficiency and generalization performance on high-dimensional data with limited samples while substantially reducing the number of model parameters;
2. An efficient manifold regularization solution algorithm is designed. Addressing the reliance on deep iterative training and high computational costs in existing manifold learning methods, this paper constructs a manifold regularization term based on an Anchor Graph. By embedding the global geometric constraints of data into the model as prior knowledge, it guides the output weight matrix to strictly follow the physical consistency of the industrial process during the reconstruction of coupling relationships. By deriving its reduced rank approximation form within the BLS framework, the graph Laplacian calculation is converted into efficient matrix algebraic operations, thereby achieving an analytical solution for output weights containing manifold constraints without iteration;
3. Experiments based on industrial datasets demonstrate that CI-GBLS exhibits superior performance in terms of prediction accuracy and training efficiency compared to existing deep learning methods and mainstream BLS variants.

The remainder of this paper is organized as follows: Section 2 reviews the standard BLS method and manifold regularization theory based on the Laplacian matrix. Section 3 introduces the CI-GBLS model and analyzes its complexity. Section 4 conducts experiments to evaluate the model’s performance. Finally, conclusions are drawn in Section 5.

## 2. Basic Theories and Methods

### 2.1. Broad Learning System

The Broad Learning System (BLS), proposed by Chen and Liu [17], is an efficient incremental learning algorithm built upon the Random Vector Functional Link Neural Network (RVFLNN). Unlike Deep Neural Networks (DNNs) that extract abstract features by increasing network depth, BLS adopts a flat network architecture, enhancing the model’s nonlinear mapping capability by laterally expanding feature nodes and enhancement nodes. Due to its extremely high training efficiency and incremental learning capability, this architecture has garnered widespread attention in the field of industrial soft sensors.

In a typical soft sensor task, given a training dataset $X\in {\mathbb{R}}^{N\times M}$ consisting of N training samples, where M denotes the dimensionality of input process variables; the corresponding output target matrix is $Y\in {\mathbb{R}}^{N\times C}$, where C represents the dimensionality of output variables. To map the original input data into a high-dimensional feature space, BLS first employs linear random mapping to generate feature nodes. Assuming the network is configured with n groups of feature mappings, each containing k nodes, the calculation of the i-th group of feature nodes $Z_{i}\in {\mathbb{R}}^{N\times k}$ is defined in Equation (1):

(1) $$Z_{i}=\phi_{i}\left(XW_{ei}+\beta_{ei}\right),\;i=1,2,\ldots ,n$$

In the above equation, the weight matrix $W_{ei}$ and the corresponding bias vector $\beta_{ei}$ are randomly generated during initialization and remain fixed during subsequent training processes, and $\phi_{i}\left(\cdot \right)$ is the feature mapping function. All feature nodes are concatenated into a feature matrix $Z^{n}=\left[Z_{1},\ldots ,Z_{n}\right]\in {\mathbb{R}}^{N\times nk}$.

To capture nonlinear characteristics of the data, the feature matrix $Z^{n}$ is mapped via a nonlinear activation function $\psi \left(\cdot \right)$ into m groups of enhancement nodes. Assuming each group contains q enhancement nodes, the j-th group of enhancement nodes $H_{j}\in {\mathbb{R}}^{N\times q}$ is calculated as shown in Equation (2):

(2) $$H_{j}=\psi \left(Z^{n}W_{hj}+\beta_{hj}\right),\;j=1,\ldots ,m$$

Here, $W_{hj}$ and $\beta_{hj}$ are the randomly generated weights and biases connecting the feature layer to the enhancement layer, respectively. All enhancement nodes are concatenated into an enhancement matrix $H^{m}=\left[H_{1},\ldots ,H_{m}\right]\in {\mathbb{R}}^{N\times mq}$. By combining the feature nodes and enhancement nodes, the system state matrix $A=\left[Z^{n}|H^{m}\right]\in {\mathbb{R}}^{N\times \left(nk+mq\right)}$ is formed. The output weight matrix $W\in {\mathbb{R}}^{\left(nk+mq\right)\times C}$ of BLS is obtained by solving the ridge regression problem in Equation (3):

(3) $$\underset{W}{\arg\min}\left({\left\|Y-AW\right\|}_{F}^{2}+\lambda {\left\|W\right\|}_{F}^{2}\right)$$

where ${\left\|\cdot \right\|}_{F}$ denotes the Frobenius norm, and λ>0 is the regularization coefficient used to control model complexity. The analytical solution to this problem is:

(4) $$W={\left(\lambda I+A^{T}A\right)}^{-1}A^{T}Y$$

where I is the identity matrix. Although the analytical solution grants BLS extremely high training efficiency, the feature construction in standard BLS relies entirely on random weights, lacking adaptability to data distribution. Furthermore, the fully connected input approach is prone to introducing redundant noise when handling high-dimensional industrial data.

### 2.2. Graph Laplacian-Based Manifold Regularization

Manifold learning theory posits that although observed data reside in a high-dimensional Euclidean space, they typically lie on an underlying low-dimensional manifold structure. In soft sensor modeling, this implies that the local geometric structure of the data contains crucial predictive information. Specifically, if two samples $x_{p}$ and $x_{q}$ are adjacent in the intrinsic geometric structure of the input space, according to the Manifold Smoothness Assumption, their predicted outputs ${\hat{y}}_{p}$ and ${\hat{y}}_{q}$ should also remain similar [32].

To enforce such geometric constraints during modeling, a manifold regularization framework based on the Graph Laplacian is commonly adopted. The first step of this approach involves constructing an adjacency graph that discretely represents the data manifold structure. The most widely used construction strategy is the k-nearest neighbors (k-NN) algorithm, which retains only the connections between each sample and its k closest neighbors, ensuring sparsity and locality of the graph. Formally, a binary adjacency matrix $G\in {\left\{0,1\right\}}^{N\times N}$ is defined as:

(5) $$G_{pq}=\left\{\begin{array}{ll} 1, & \mathrm{if}\;x_{p}\in N_{k}\left(x_{q}\right)\;\mathrm{or}\;x_{q}\in N_{k}\left(x_{p}\right)\\ 0, & \mathrm{otherwise} \end{array}\right.$$

where $N_{k}\left(x_{i}\right)$ denotes the set of k nearest neighbors of sample $x_{i}$. Based on this topology, the affinity between samples is quantified using a Gaussian kernel function, yielding a weight matrix $S\in {\mathbb{R}}^{N\times N}$:

(6) $$S_{pq}=\left\{\begin{array}{ll} G_{pq}\cdot \exp\left(-\frac{{\left\|x_{p}-x_{q}\right\|}^{2}}{2\sigma^{2}}\right), & \mathrm{if}\;G_{pq}=1\\ 0, & \mathrm{otherwise} \end{array}\right.$$

where σ is the kernel width parameter. On this basis, the manifold regularization term $J_{MR}$ is defined as the weighted sum of squared differences in predicted outputs over all neighboring sample pairs. Using the Laplacian matrix L=D−S, where D is the degree matrix with diagonal elements $D_{pp}=\sum_{q}S_{pq}$, the regularization term can be expressed in a compact matrix trace form:

(7) $$J_{MR}=\frac{1}{2}{\sum_{p,q}S_{pq}\left\|{\hat{y}}_{p}-{\hat{y}}_{q}\right\|}^{2}=Tr\left({\hat{Y}}^{T}L\hat{Y}\right)$$

Incorporating this regularization term into the objective function of BLS leads to the following joint optimization problem with manifold constraints:

(8) $$J\left(W\right)={\left\|Y-AW\right\|}_{F}^{2}+\lambda {\left\|W\right\|}_{F}^{2}+\beta Tr\left(W^{T}A^{T}LAW\right)$$

where β is a hyperparameter controlling the strength of the manifold constraint. Although this framework effectively leverages data geometry to enhance generalization performance, constructing the k-NN graph and computing the Laplacian matrix require calculating pairwise distances among all samples, resulting in time and space complexity as high as $O\left(N^{2}\right)$.

## 3. Proposed Method

**Figure 1:**
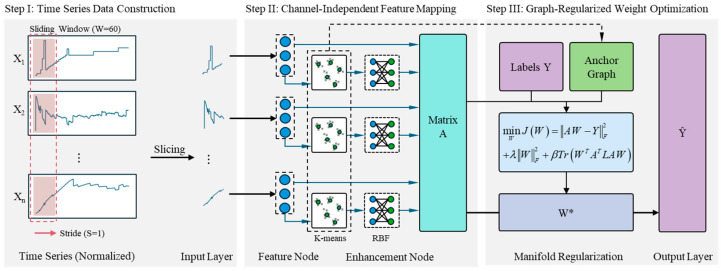
The overall framework of the proposed CI-GBLS. In this figure, the blue circles represent the feature nodes, the gray crosses denote the mapped data points, and the green circles indicate the cluster centers (anchors) obtained via the K-means algorithm.

To address the prevalent challenges of strong variable coupling and nonlinear time-varying dynamics in complex industrial process data, this paper proposes a Channel-Independent Anchor Graph-Regularized Broad Learning System (CI-GBLS). The overall computational architecture of the model is illustrated in Figure 1.

To achieve decoupled modeling of temporal dynamics and spatial coupling in multivariate inputs, CI-GBLS first introduces a Channel-Independent (CI) mechanism, which decouples multivariate inputs into independent feature channels for parallel mapping. Subsequently, the model utilizes K-Means adaptive anchors to construct RBF mappings, enhancing nonlinear fitting capability while capturing the global structure of the data. Finally, by deriving a low-rank analytical solution of the manifold regularization term based on anchor graph theory, the model achieves improvements in both accuracy and computational efficiency.

### 3.1. Principle and Implementation of CI-GBLS

For an industrial soft sensor task involving N training samples and M process variables, let the input data be denoted as $X\in {\mathbb{R}}^{N\times M}$ and the corresponding output targets as $Y\in {\mathbb{R}}^{N\times C}$. CI-GBLS adopts a Channel Independence (CI) strategy. Specifically, the input data are treated as M independent univariate time series $x_{i}\in {\mathbb{R}}^{N\times 1}\left(i=1,\ldots ,M\right)$, and physically isolated feature mapping channels are constructed for each variable. Within the i-th channel, the input data are transformed into feature nodes $Z_{i}$ via linear random mapping:

(9) $$Z_{i}=\phi \left(x_{i}W_{e,i}+\beta_{e,i}\right),\;i=1,\ldots ,M$$

where $W_{e,i}$ and $\beta_{e,i}$ are weight and bias parameters unique to the i-th channel, and $\phi \left(\cdot \right)$ denotes the activation function. To maintain computational efficiency and avoid excessive search space dimensionality, all independent channels are configured with an identical set of hyperparameters, thereby ensuring that all variables possess sufficient and consistent feature extraction capabilities to mine their intrinsic temporal characteristics. Feature nodes from all channels are then concatenated into the overall feature matrix $Z_{f}=\left[Z_{1},\ldots ,Z_{M}\right]$.

To further enhance the model’s capability in fitting complex nonlinear dynamics, an anchor-based Radial Basis Function (RBF) mapping mechanism is introduced in the enhancement layer. For the i-th feature channel $Z_{i}$, $m_{k}$ channel-specific anchor points $U_{i}=\left\{u_{i,1},\ldots ,u_{i,m_{k}}\right\}$ are adaptively clustered using the K-Means algorithm, avoiding the high computational burden associated with constructing a full-sample similarity matrix, thereby maintaining the linear complexity of the algorithm. Subsequently, the RBF kernel distance between the channel features $Z_{i}$ and its anchor points $U_{i}$ is computed to generate the enhancement nodes $H_{i}$ for the i-th channel:

(10) $$H_{i,jk}=\exp\left(-\gamma {\left\|z_{i,j}-u_{i,k}\right\|}^{2}\right)$$

Finally, the enhancement nodes from all channels are concatenated into $H=\left[H_{1},\ldots ,H_{M}\right]$. The feature nodes and enhancement nodes are combined to form the final system state matrix $A=\left[Z_{f}|\xi H\right]$, where ξ is a scaling factor that regulates the contribution ratio between feature and enhancement nodes. Based on the constructed global system state matrix A, the model performs explicit reconstruction of multivariate spatiotemporal information in the output layer. The prediction equation Y=AW is equivalent to a global linear spanning over the decoupled feature bases:

(11) $$Y=AW=\sum_{i=1}^{M}Z_{i}W_{Z,i}+\xi \sum_{j=1}^{M}H_{j}W_{Z,j}$$

Here, the weight matrix W acts as a coupling operator, projecting the originally orthogonal univariate temporal modes into a unified output subspace through cross-channel parameter associations, thereby recovering the spatial coupling representation among variables.

To fully exploit the geometric distribution information embedded in the data, CI-GBLS introduces a manifold regularization term based on an Anchor Graph into the objective function. In contrast to conventional methods, we directly construct the topological structure using the fully concatenated enhancement matrix H, thus capturing the global manifold distribution of the data in the joint feature space.

Based on Anchor Graph Theory, we first perform row-wise normalization on H to obtain $\bar{H}$. The full-sample adjacency matrix S can be approximated by a low-rank matrix $\hat{S}=\bar{H}\Delta^{-1}{\bar{H}}^{T}$, where $\Delta \in {\mathbb{R}}^{m\times m}$ is a diagonal normalization matrix with elements $\Delta_{kk}=\sum_{j=1}^{N}{\bar{H}}_{jk}$ Accordingly, the joint optimization objective function incorporating the manifold constraint is defined as:

(12) $$\underset{W}{\min}J\left(W\right)={\left\|AW-Y\right\|}_{F}^{2}+\lambda {\left\|W\right\|}_{F}^{2}+\beta Tr\left(W^{T}A^{T}LAW\right)$$

where $L=I-\hat{S}$ is the random-walk normalized Laplacian matrix reconstructed from the anchor graph. By leveraging the low-rank approximation, the manifold regularization term $A^{T}LA$ can be efficiently derived and transformed into:

(13) $$A^{T}LA=A^{T}\left(I-\bar{H}\Delta^{-1}{\bar{H}}^{T}\right)A=A^{T}A-\left(A^{T}\bar{H}\right)\Delta^{-1}\left({\bar{H}}^{T}A\right)$$

This transformation shifts the computational bottleneck from quadratic complexity in the sample dimension to matrix multiplications involving the feature and anchor dimensions. By differentiating the objective function and setting the derivative to zero, along with the above derivation, we ultimately obtain the analytical solution for the output weights in CI-GBLS:

(14) $$\left(\left(1+\beta \right)A^{T}A-\beta \left(A^{T}\bar{H}\right)\Delta^{-1}\left({\bar{H}}^{T}A\right)+\lambda I\right)W=A^{T}Y$$

The coefficient matrix on the left-hand side of Equation (14) can be rewritten as $(A^{T}A+\lambda I)+\beta \left(A^{T}A-(A^{T}\bar{H})\Delta^{-1}({\bar{H}}^{T}A)\right)$. Here, the first part, $\left(A^{T}A+\lambda I\right)$, corresponds to standard ridge regression; the second part, $\beta \left(A^{T}A-(A^{T}\bar{H})\Delta^{-1}({\bar{H}}^{T}A)\right)$, introduces an anchor graph-based manifold regularization constraint. When β is small, the analytical solution is dominated by the first part, and the model focuses on closely fitting the data. Conversely, when β is large, the manifold constraint of the second part is strengthened, forcing the output weights to conform to the global geometric structure to suppress overfitting; however, an excessively large β may lead to over-smoothing.

This solution requires no gradient-based iteration and yields the globally optimal solution through direct linear algebraic operations, significantly reducing computational cost while ensuring manifold consistency in the learned representation.

### 3.2. Computational Complexity Derivation and Analysis

To rigorously verify the computational efficiency of the proposed method mathematically, this section presents a full-process derivation of the time complexity of CI-GBLS based on fundamental principles of matrix operations and compares it with BLS, RBF-BLS without Channel Independence (RBF-BLS w/o CI), and conventional Manifold-Regularized BLS (Lap-BLS). For ease of analysis, the following variables are defined: let N denote the number of training samples, M the dimensionality of input variables, $L_{f}$ the number of feature nodes, $L_{e}$ the number of enhancement nodes, and m the number of anchor points. In the CI-GBLS architecture proposed in this paper, enhancement nodes are generated from RBF distances between feature nodes and anchor points; therefore, the total number of anchor points m equals the total number of enhancement nodes $L_{e}$. Let $L=L_{f}+L_{e}$ denote the total number of network nodes, and I denote the average number of iterations in the K-Means clustering algorithm. Industrial process data typically satisfy N≫L>m>M.

In the feature mapping stage, computing $Z=\phi \left(XW_{e}+\beta_{e}\right)$, BLS, RBF-BLS, and Lap-BLS adopt fully connected mappings. Computing $N\times L_{f}$ elements, each involving a dot product of M-dimensional vectors, results in complexity $O\left(N\cdot M\cdot L_{f}\right)$. CI-GBLS employs a Channel-Independent (CI) strategy, decoupling the input space into M independent channels. The computational load per channel is $N\cdot 1\cdot \left(L_{f}/M\right)$, and summing over M channels yields a total complexity of $O\left(N\cdot L_{f}\right)$.

In the enhancement node generation stage, computing the enhancement nodes H, BLS and Lap-BLS use one-time random projection, resulting in complexity $O\left(N\cdot L_{f}\cdot L_{e}\right)$. RBF-BLS constructs the RBF mapping via global K-Means clustering, an iterative process requiring I iterations. Each iteration computes Euclidean distances from N samples to m anchor points in $L_{f}$-dimensional space, leading to complexity $O\left(I\cdot N\cdot m\cdot L_{f}\right)$. CI-GBLS employs Channel-Independent K-Means clustering. It also requires I iterations, but the clustering is confined to low-dimensional subspaces. Per channel, each iteration involves $N\cdot \left(m/M\right)\cdot \left(L_{f}/M\right)$ operations, and summing over M channels gives a total complexity of $O\left(\frac{I\cdot N\cdot m\cdot L_{f}}{M}\right)$.

In the manifold regularization stage, Lap-BLS constructs a full-sample N×N adjacency matrix, with graph construction complexity $O\left(N^{2}L_{f}\right)$, while the regularizer multiplication $A^{T}LA$ involves N×N matrix operations, yielding complexity $O\left(N^{2}L\right)$. Thus, the overall complexity of the manifold regularization stage is approximately $O\left(N^{2}L\right)$. In contrast, CI-GBLS reuses the RBF distances already computed in the enhancement layer, incurring zero additional cost for graph construction. By leveraging low-rank approximation via the anchor-point graph, the regularizer multiplication only involves N×m matrix operations, resulting in complexity $O\left(NmL\right)$.

In the model-solving stage, all algorithms compute the Hessian matrix $A^{T}A$ and its inverse, with complexity approximately $O\left(NL^{2}\right)$. Summing the complexities across all stages yields the detailed comparison presented in Table 1.

In summary, CI-GBLS achieves linear time complexity throughout the entire process by means of structural channel decoupling and algorithmic anchor-point approximation. This enables the proposed method to efficiently handle large-scale, high-dimensional industrial process data while preserving manifold geometric constraints.

## 4. Experiments and Analysis

This chapter comprehensively evaluates the effectiveness of the proposed Channel-Independent Anchor Graph-Regularized Broad Learning System (CI-GBLS) through soft sensor experiments based on the Industrial-scale penicillin simulation (IndPenSim). First, the dataset characteristics, preprocessing procedures, and experimental parameter settings are introduced. Subsequently, through comparisons with mainstream BLS variants and state-of-the-art deep learning methods, the analysis focuses on the dual advantages of CI-GBLS in terms of prediction accuracy and computational efficiency. Finally, ablation studies and parameter sensitivity analyses are conducted to further validate the contribution and robustness of the model’s core components.

### 4.1. Experimental Settings

#### 4.1.1. Data Description and Preprocessing

The experimental data originates from the IndPenSim simulation platform developed by Goldrick et al. [33,34], designed to provide a high-fidelity simulation of a 100,000-L industrial-scale penicillin fermentation process. The dataset encompasses all available process data from 100 batches, incorporating diverse control strategies and varying batch durations to represent typical biopharmaceutical production facilities. Specifically, batches 1–30 utilize recipe-based control, batches 31–60 are operator-controlled, batches 61–90 implement an Advanced Process Control (APC) scheme based on Raman spectroscopy, and batches 91–100 contain faults leading to process deviations. The process exhibits typical characteristics of multivariate coupling, nonlinear dynamics, and batch-to-batch variability, with individual batches typically lasting 230 h.

In this study, penicillin concentration is selected as the Key Quality Attribute (KQA) to be predicted, and faulty batches were excluded. The input feature set encompasses high-frequency sampled process variables with a sampling interval of 12 min, including manipulated variables such as substrate flow rate and phenylacetic acid flow rate, as well as process variables like temperature, pH, dissolved oxygen, and off-gas analysis.

To address the prevalent challenges of measurement delay and multi-rate sampling in industrial data, this paper designs the following rigorous data preprocessing pipeline:

1. Physical Alignment: Considering the approximate 4-h analytical delay in offline assays, a time-shifting operation was applied to the target variable to ensure temporal consistency between inputs and outputs;
2. Continuous Label Reconstruction: Given that the quality variables were sampled every 12 h, the data exhibited severe multi-rate characteristics. Considering the physical properties of large inertia and continuity inherent in the fermentation process, quality variables typically exhibited a slowly varying trend. To achieve online real-time prediction, it was necessary to align the sparse quality data with the high-frequency process data. Therefore, linear interpolation combined with boundary padding was employed as a reasonable approximation of the quality trend during unobserved periods. This approach effectively filled the dynamic information gaps within sampling intervals, guiding the model to capture the principal evolutionary trends of the quality variables, thereby fully exploiting the high-frequency dynamic information of process variables for supervised learning;
3. Stratified Data Splitting: Considering the dataset includes multiple control modes, a batch-based stratified splitting strategy was adopted. 80% of the batches were allocated to the training set and 20% to the test set, ensuring that both sets cover different operating condition distributions to effectively evaluate the model’s generalization capability.

Finally, a time-series feature matrix is constructed using a sliding window technique. All variables underwent Min-Max normalization, where normalization parameters were calculated exclusively on the training set to simulate a realistic industrial deployment environment.

#### 4.1.2. Implementation Details

All experiments were conducted on a workstation equipped with an Intel Core i7-10700F CPU (Intel Corporation, Santa Clara, CA, USA), NVIDIA T600 GPU (NVIDIA Corporation, Santa Clara, CA, USA), and 16 GB of RAM. To eliminate the influence of random initialization, all models were run independently 5 times, and the mean and standard deviation of evaluation metrics are reported.

To ensure fairness and validity in comparative experiments, we performed tailored adaptations to the network architectures of state-of-the-art models originally designed for multivariate long-term time series forecasting tasks—such as DLinear [29] and PatchTST [30]—to make them applicable to soft sensor regression tasks.

Specifically, we retained the core temporal feature extraction mechanisms of each model: DLinear continues to employ the Trend-Seasonal Decomposition strategy, while PatchTST maintains its Patch embedding and Transformer encoder architecture. However, to transform the model output from multivariate future sequences to a current estimate of a single quality variable, we introduced a Global Regression Head to replace the original forecasting head. For DLinear, after linear projection along the time dimension, a Channel Mixing Layer was added to perform a weighted aggregation of features from all independent channels to output the final prediction. For PatchTST, we flattened the latent representations across all channels and patches and mapped them non-linearly via a Multilayer Perceptron (MLP) containing Batch Normalization and Dropout. These modifications ensure that the baseline models can leverage their advanced temporal modeling capabilities while effectively capturing the strong inter-variable coupling characteristics required for a soft sensor.

#### 4.1.3. Evaluation Metrics

To quantitatively evaluate the predictive performance of CI-GBLS and the comparative models, this paper adopts three standard statistical metrics: Root Mean Square Error (RMSE), Mean Absolute Error (MAE), and the Coefficient of Determination ($R^{2}$). RMSE and MAE are utilized to measure the deviation between predicted and actual values, with lower values indicating higher accuracy. Their definitions are as follows:

(15) $$RMSE=\sqrt{\frac{1}{N_{test}}\sum_{i=1}^{N_{test}}{\left(y_{i}-{\hat{y}}_{i}\right)}^{2}}$$

(16) $$MAE=\frac{1}{N_{test}}\sum_{i=1}^{N_{test}}\left|y_{i}-{\hat{y}}_{i}\right|$$

where $N_{test}$ denotes the number of test samples, $y_{i}$ represents the true quality value of the i-th sample, and ${\hat{y}}_{i}$ denotes the corresponding predicted value generated by the model. Furthermore, $R^{2}$ is used to assess the goodness of fit, representing the proportion of variance in the dependent variable that can be explained by the independent variables. An $R^{2}$ value closer to 1 indicates a superior model fit:

(17) $$R^{2}=1-\frac{\sum_{i=1}^{N_{test}}{\left(y_{i}-{\hat{y}}_{i}\right)}^{2}}{\sum_{i=1}^{N_{test}}{\left(y_{i}-\bar{y}\right)}^{2}}$$

where $\bar{y}$ is the mean of the observed data. Beyond prediction accuracy, computational efficiency is also a critical factor for industrial deployment. Therefore, we further recorded the Training Time of each model for comparative analysis.

### 4.2. Performance Comparison and Analysis

To validate the effectiveness of the proposed method, we compared CI-GBLS with five representative baseline methods, including standard BLS variants (BLS, AKBLS) and advanced deep learning models (LSTM, DLinear, PatchTST).

To ensure fairness in the comparative experiments and fully exploit the performance potential of each model, we constrained the total number of nodes for the BLS-based shallow models to balance computational cost against performance, ensuring that the model scale remained comparable to that of the baseline models. A grid search strategy was employed to optimize the regularization coefficient λ, the RBF kernel width γ, and the manifold regularization coefficient β. Specifically, λ and γ were searched over the ranges $\left[10^{-4},10\right]$ and $\left[10^{-4},1\right]$, respectively, following a log-uniform distribution; while β was searched over the discrete set {0,0.01,0.05}. To examine model behavior under extreme conditions, a broader search range was adopted for β in the subsequent sensitivity analysis. In practice, 20% of the training set was reserved as a validation set. All parameter combinations within the predefined search space were exhaustively evaluated, and the configuration yielding the minimum Root Mean Square Error (RMSE) on the validation set was selected as the final choice. To reduce the search space dimensionality, the scaling factor ξ was empirically fixed at 0.5, referencing the original BLS framework proposed by Chen et al. [17] and based on observations from our preliminary experiments.

For deep learning comparison models, considering their high computational training cost, we adopted commonly recommended configurations from the literature and fine-tuned them based on the validation set. All deep models were trained using the Adam optimizer with a maximum of 200 epochs, and an Early Stopping mechanism was implemented to prevent overfitting.

The final optimal hyperparameter configurations for all models are summarized in Table 2.

Table 3 details the performance of each model on the test set. As shown in Table 3, CI-GBLS outperforms all competing methods across all accuracy metrics. Compared to BLS, the RMSE is reduced by approximately 23%, demonstrating the effectiveness of incorporating manifold regularization and a Channel-Independent structure. Notably, although PatchTST and DLinear exhibit strong performance in time series forecasting, their performance is constrained on this industrial dataset. The relatively large standard deviation observed in the training time of deep learning models, particularly PatchTST, is primarily attributed to the early stopping mechanism, as different random initializations lead to significant variations in the number of epochs required for convergence.

**Figure 2:**
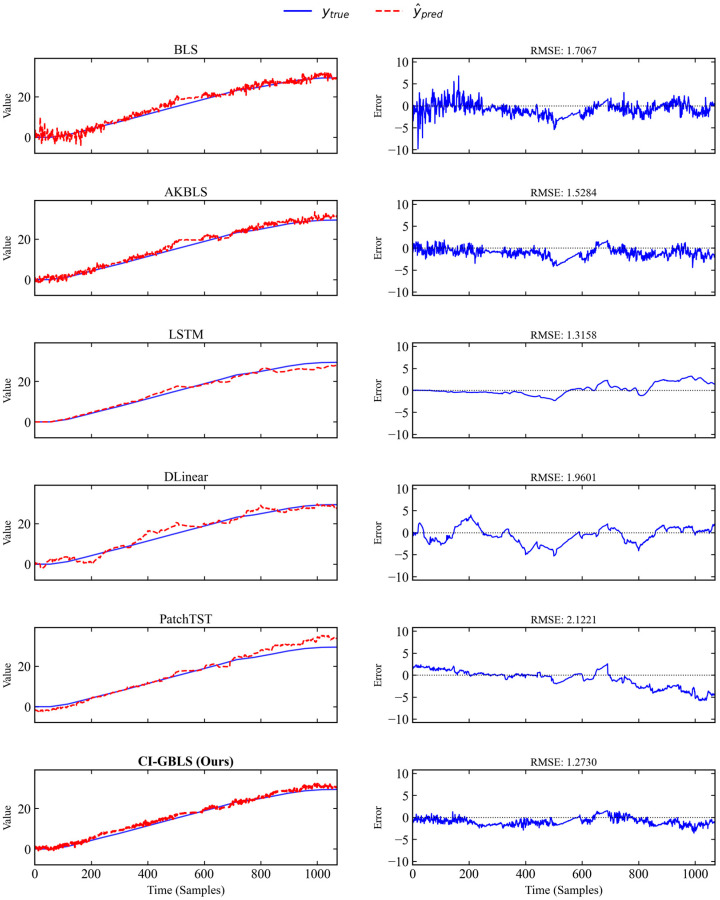
Prediction curves of different models on a representative test batch.

To visually illustrate the prediction performance, Figure 2 plots the prediction curves of each model on a typical test batch. The left side of Figure 2 shows the prediction results, while the right side displays the error curves between predictions and ground truth. As shown in Figure 2, the red dashed line representing CI-GBLS in the bottom row exhibits the highest alignment with the true values (blue solid line), particularly during the mid-to-late fermentation stages. The CI-GBLS prediction curve remains closely aligned with the true values, demonstrating a sharp dynamic capture capability. The Channel-Independent design and manifold regularization reduce prediction noise, resulting in smaller fluctuations in the error curve compared to other models. In contrast, the three deep learning models exhibit increasing errors during the mid-to-late phases. This may be attributed to the fact that Transformer architectures typically require massive amounts of data to unleash their advantages in long-sequence modeling, whereas on industrial datasets like IndPenSim—characterized by relatively limited samples and stronger dependence on local manifold consistency—overly complex attention mechanisms can easily lead to overfitting.

**Figure 3:**
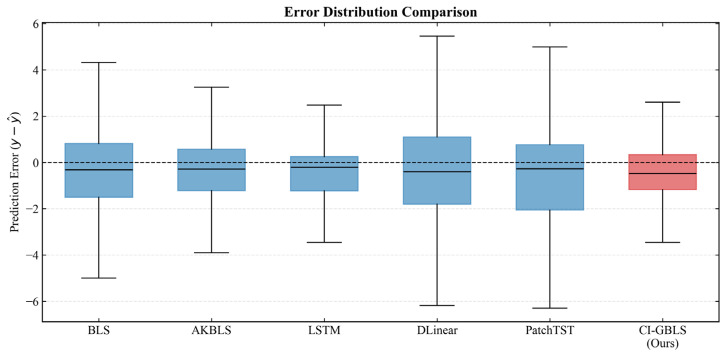
Boxplot of prediction error distribution.

**Figure 4:**
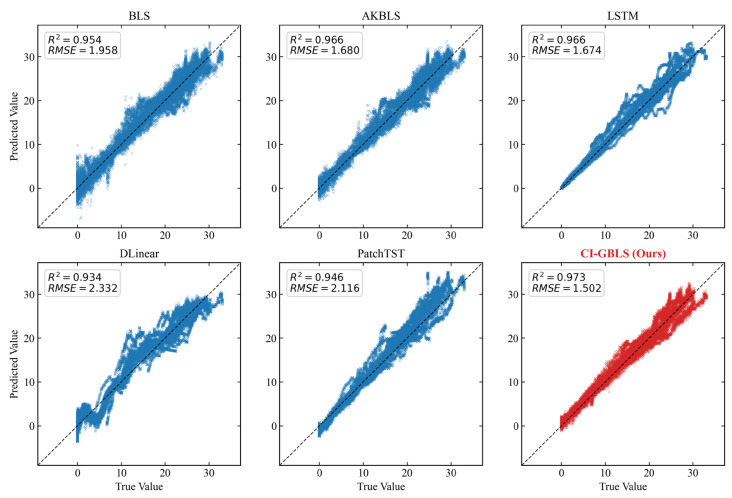
Regression scatter plots of predicted vs. true values (from the median performance run).

The error box plot in Figure 3 further verifies the robustness of the models. The median line of CI-GBLS is nearly centered within the box, indicating a well-balanced symmetry in error distribution. Although the entire box exhibits a slight offset from the zero line, CI-GBLS demonstrates a more concentrated error distribution range with minimal prediction error variance, reflecting exceptional stability. The regression analysis plot in Figure 4 corroborates this observation: the predicted points of CI-GBLS are most tightly clustered around the diagonal line, achieving an $R^{2}$ of up to 0.973.

Furthermore, to validate the reliability of the performance improvement, we conducted a statistical significance test (Welch’s *t*-test). The results indicate that the performance difference between CI-GBLS and the second-best baseline model (AKBLS) is highly statistically significant (*p* < 0.001), confirming that the improvement in the model error distribution is not due to chance.

**Figure 5:**
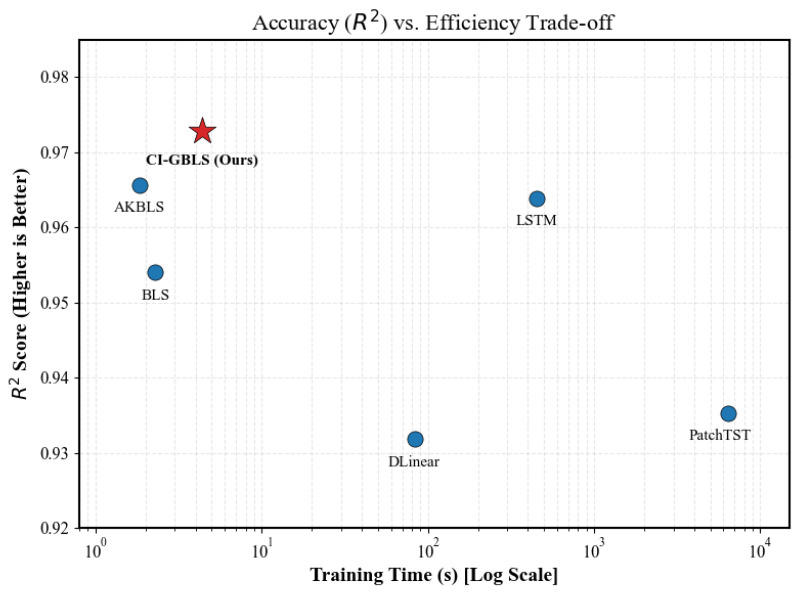
Accuracy (*R*^2^) vs. Efficiency trade-off analysis.

To intuitively assess the trade-off between accuracy and speed among models, we plot each model’s coefficient of determination ($R^{2}$) against its training time in Figure 5. In this figure, the horizontal axis represents training time on a logarithmic scale—values farther to the left indicate faster training—while the vertical axis denotes the $R^{2}$ score, with higher values indicating greater accuracy. As shown in Figure 5, the proposed CI-GBLS method (red star) occupies the optimal top-left region. Specifically, in terms of accuracy, CI-GBLS is positioned at the very top of the chart ($R^{2}$ = 0.9728), indicating its superior ability to explain industrial process dynamics, surpassing even complex deep learning models. In terms of efficiency, CI-GBLS is located at the far left of the chart, requiring only 4.36 s for training. In contrast, PatchTST is positioned at the far right, consuming as much as 6365 s.

### 4.3. Ablation Study and Sensitivity Analysis

**Figure 6:**
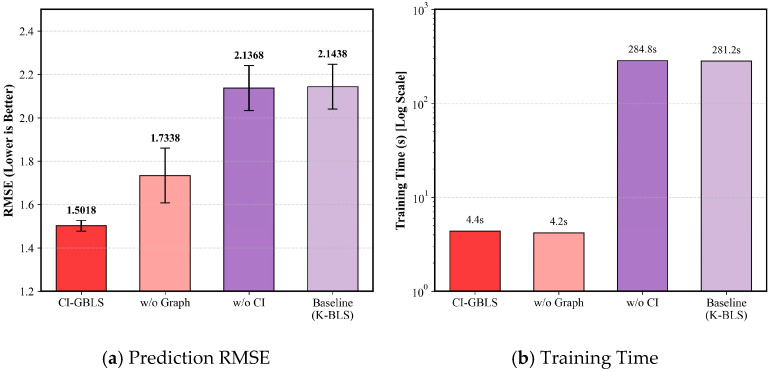
Impact of different modules on (**a**) Prediction RMSE and (**b**) Training Time.

To validate the contribution of the core components in the proposed method, we conducted ablation experiments, comparing the full model (CI-GBLS), the model without anchor graph regularization (w/o Graph), the model without the Channel Independence structure (w/o CI), and the baseline model (K-BLS). The ablation results are presented in Table 4 and Figure 6. Comparing Proposed with w/o CI (Figure 6b), upon removing the CI structure, the model training time surged from 4.36 s to 284.81 s, while the RMSE deteriorated from 1.50 to 2.13. This demonstrates that the CI strategy successfully reduces computational complexity substantially by decoupling input variables. Comparing Proposed with w/o Graph (Figure 6a), the introduction of anchor graph regularization further reduces RMSE. This indicates that the anchor-based manifold constraint effectively captures the intrinsic geometric structure of the data, thereby enhancing the model’s generalization capability.

Notably, the ablation results further reveal that the performance degradation caused by removing the CI structure was significantly more severe than that caused by removing the manifold regularization term; the RMSE rose from 1.50 to 2.13, whereas removing the graph regularization term only resulted in an increase to 1.73. This phenomenon underscores that the CI strategy is the primary factor contributing to the success of CI-GBLS. The underlying reason is that traditional fully connected structures attempt to simultaneously extract multivariate spatial correlations and univariate temporal features. Such high coupling hinders the model from fully capturing the intrinsic temporal characteristics of each variable while concurrently introducing noise interference. In contrast, in CI-GBLS, variables are first processed within independent channels to mine their individual evolutionary patterns, followed by global information aggregation at the output layer. This strategy effectively captures the temporal features of variables during the feature extraction stage, thereby demonstrating enhanced robustness when handling industrial data characterized by multi-rate sampling.

**Figure 7:**
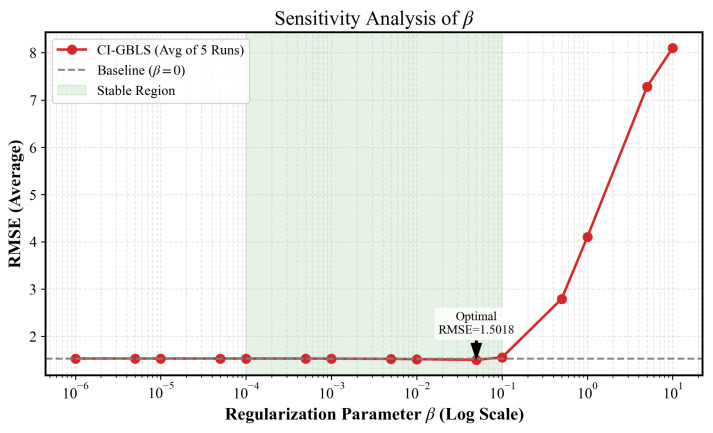
Sensitivity analysis of the regularization parameter β.

In the objective function of CI-GBLS, the regularization coefficient β governs the strength of the manifold consistency constraint. We traversed β values over a wide range of $\left[10^{-6},10^{1}\right]$, with results shown in Figure 7. CI-GBLS exhibits exceptional robustness. Within the stable interval marked in green, the RMSE curve remains flat and at its lowest level. This implies that in practical industrial deployment, users can achieve optimal performance without the need for meticulous parameter fine-tuning. However, when β>1, the error increases significantly. According to the analytical solution in Equation (14), the constraining effect of β on the output weights W follows an inversely proportional decay law, i.e., $\left\|W\right\|\propto 1/\left(1+\beta \right)$. When β reaches the critical point of 1, the magnitude of W decreases by approximately 50%, thereby inducing an over-smoothing effect. This forces the model into an underfitting state, causing it to lose the ability to capture the high-frequency dynamics of industrial processes, resulting in a sharp increase in RMSE.

## 5. Conclusions

This paper proposes a Channel-Independent Anchor Graph-Regularized Broad Learning System (CI-GBLS) to address the challenges of strong coupling noise interference and nonlinear time-varying dynamics in high-dimensional multivariate data for industrial soft sensors. This method incorporates the Channel Independence (CI) strategy into the Broad Learning framework. By decoupling input variables and constructing independent feature mapping channels, CI-GBLS effectively blocks noise propagation among irrelevant variables, significantly reducing the risk of overfitting when processing high-dimensional industrial data. Furthermore, the introduction of anchor graph-based manifold regularization further guarantees the model’s generalization robustness. By repurposing the cluster centers of RBF enhancement nodes as graph anchors, the method successfully embeds the nonlinear manifold structure of the data into the learning objective, forcing the model output to strictly adhere to the data’s intrinsic geometric distribution. This geometric consistency constraint not only smooths out local prediction fluctuations caused by measurement noise but also significantly enhances the model’s robustness against operational variations across different fermentation batches. Experimental results on the IndPenSim benchmark dataset demonstrate that CI-GBLS outperforms existing deep learning methods and mainstream BLS variants in prediction accuracy while maintaining the advantage of second-level convergence in training efficiency. With extremely high prediction accuracy and negligible computational overhead, CI-GBLS offers a highly promising solution for real-time online monitoring in large-scale industrial processes.

Despite the outstanding performance of CI-GBLS in offline modeling, several research directions remain to be explored. First, industrial processes often exhibit time-varying drift characteristics. Although the Broad Learning System possesses incremental learning capabilities, further investigation is needed on how to achieve low-cost online updates of the inverse matrix and anchor structures after incorporating anchor graph regularization terms. Second, the current anchors are generated via fixed K-Means clustering; future work may consider introducing dynamic anchor selection strategies to allow anchor distributions to more accurately track the manifold evolution of process data.

## Figures and Tables

**Table 1:** Exact Comparison of Computational Complexity.

Algorithm	Feature Mapping	Enhancement Generation	Regularization	Model Solving	Total Complexity
BLS	$O(NML_{f})$	$O\left(NL_{f}L_{e}\right)$		$O\left(NL^{2}\right)$	$O\left(N\left(ML_{f}+L_{f}m+L^{2}\right)\right)$
RBF-BLS	$O(NML_{f})$	$O\left(INmL_{f}\right)$		$O\left(NL^{2}\right)$	$O\left(N\left(ML_{f}+IL_{f}m+L^{2}\right)\right)$
Lap-BLS	$O(NML_{f})$	$O\left(NL_{f}L_{e}\right)$	$O(N^{2}L)$	$O\left(NL^{2}\right)$	$O\left(N^{2}L\right)$
CI-GBLS	$O(NL_{f})$	$O\left(\frac{INmL_{f}}{M}\right)$	$O\left(NmL\right)$	$O\left(NL^{2}\right)$	$O\left(N\left(L_{f}+\frac{ImL_{f}}{M}+mL+L^{2}\right)\right)$

**Table 2:** Detailed hyperparameter settings for all competitive models.

Model Type	Model Name	Key Hyperparameters
Proposed	CI-GBLS	Structure: $L_{f}=180$ (10 per var), $L_{e}=180$ (10 per var) Params: ξ=0.5, $\lambda =10^{-3}$, γ=0.1, β=0.05
BLS Baselines	BLS	Structure: $L_{f}=180$, $L_{e}=180$ Params: ξ=0.5, $\lambda =10^{-2}$
	AKBLS	Structure: $L_{f}=180$, $L_{e}=180$ Params: ξ=0.5, λ=1, $\gamma =10^{-4}$
Deep Learning	PatchTST	Structure: $D_{model}=128$, Heads=8, layers=2, Patch=12 Train: Batch=128, $LR=10^{-3}$, Dropout=0.1
	DLinear	Train: Batch=128, $LR=10^{-3}$
	LSTM	Structure: Hidden=128, layers=2 Train: Batch=128, $LR=10^{-3}$

**Table 3:** Performance comparison of different methods on the IndPenSim dataset (Mean ± Std over 5 runs).

Methods	RMSE	MAE	*R* ^2^	Train Time (s)
CI-GBLS (Ours)	1.5018 ± 0.0242	1.1354 ± 0.0219	0.9728 ± 0.0009	4.36 ± 0.17
AKBLS	1.6896 ± 0.0179	1.2686 ± 0.0140	0.9656 ± 0.0007	1.83 ± 0.13
LSTM	1.7265 ± 0.1134	1.1994 ± 0.0885	0.9639 ± 0.0048	451.01 ± 95.01
BLS	1.9539 ± 0.0389	1.4997 ± 0.0373	0.9540 ± 0.0018	2.29 ± 0.31
PatchTST	2.2728 ± 0.4514	1.7891 ± 0.4140	0.9353 ± 0.0255	6365.16 ± 1724.64
DLinear	2.3753 ± 0.1102	1.8622 ± 0.0938	0.9318 ± 0.0064	83.11 ± 15.72

**Table 4:** Quantitative results of the ablation study (Mean ± Std over 5 runs).

Methods	RMSE	MAE	*R* ^2^	Train Time (s)
CI-GBLS	1.5018 ± 0.0242	1.1354 ± 0.0219	0.9728 ± 0.0009	4.36 ± 0.17
w/o Graph	1.7338 ± 0.1258	1.3182 ± 0.1113	0.9636 ± 0.0050	4.19 ± 0.11
w/o CI	2.1368 ± 0.1038	1.6566 ± 0.0791	0.9448 ± 0.0052	284.81 ± 2.50
K-BLS	2.1438 ± 0.1039	1.6619 ± 0.0780	0.9445 ± 0.0052	281.17 ± 1.74

## Data Availability

Publicly available datasets were analyzed in this study. The IndPenSim benchmark dataset (100 batches) used in this research can be found here: http://www.industrialpenicillinsimulation.com/ (accessed on 26 January 2026).
